# Location and Types of Treatment for Prostate Cancer After the Veterans Choice Program Implementation

**DOI:** 10.1001/jamanetworkopen.2023.38326

**Published:** 2023-10-19

**Authors:** Bradley A. Erickson, Richard M. Hoffman, Jason Wachsmuth, Vignesh T. Packiam, Mary S. Vaughan-Sarrazin

**Affiliations:** 1 Veterans Health Administration (VHA) Office of Rural Health, Iowa City Veterans Affairs Health Care System, Iowa City, Iowa; 2 Department of Urology, University of Iowa Carver College of Medicine, Iowa City; 3 Department of Internal Medicine, Division of General Internal Medicine, University of Iowa Carver College of Medicine, Iowa City; 4 VHA Office of Rural Health, Iowa City Veterans Affairs Health Care System, Center for Access and Delivery Research and Evaluation (CADRE), Iowa City, Iowa

## Abstract

**Importance:**

The Veterans Choice Program (VCP) was implemented in 2014 to help veterans gain broader access to specialized care outside of the Veterans Health Administration (VHA) facilities by providing them with purchased community care (CC).

**Objective:**

To describe the prevalence and patterns in VCP-funded purchased CC after the implementation of the VCP among veterans with prostate cancer.

**Design, Setting, and Participants:**

This cohort study used VHA administrative data on veterans with prostate cancer diagnosed between January 1, 2015, and December 31, 2018. These veterans were regular VHA primary care users. Analyses were performed from March to July 2023.

**Exposures:**

Driving distance (in miles) from residence to nearest VHA tertiary care facility. The location (VHA or purchased CC) in which treatment decisions were made was ascertained by considering 3 factors: (1) location of the diagnostic biopsy, (2) location of most of the postdiagnostic prostate-specific antigen laboratory testing, and (3) location of most of the postdiagnostic urological care encounters.

**Main Outcomes and Measures:**

The main outcome was receipt of definitive treatment and proportion of purchased CC by treatment type (radical prostatectomy [RP], radiotherapy [RT], or active surveillance) and by distance to nearest VHA tertiary care facility. Quality was evaluated based on receipt of definitive treatment for Gleason grade group 1 prostate cancer (low risk/limited treatment benefit by guidelines).

**Results:**

The cohort included 45 029 veterans (mean [SD] age, 67.1 [6.9] years) with newly diagnosed prostate cancer; of these patients, 28 866 (64.1%) underwent definitive treatment. Overall, 56.8% of patients received definitive treatment from the purchased CC setting, representing 37.5% of all RP care and 66.7% of all RT care received during the study period. Most patients who received active surveillance management (92.5%) remained within the VHA. Receipt of definitive treatment increased over the study period (from 5830 patients in 2015 to 9304 in 2018), with increased purchased CC for patients living farthest from VHA tertiary care facilities. The likelihood of receiving definitive treatment of Gleason grade group 1 prostate cancer was higher in the purchased CC setting (adjusted relative risk ratio, 1.79; 95% CI, 1.65-1.93).

**Conclusions and Relevance:**

This cohort study found that the percentage of veterans receiving definitive treatment in VCP-funded purchased CC settings increased significantly over the study period. Increased access, however, may come at the cost of low care quality (overtreatment) for low-risk prostate cancer.

## Key Points

**Question:**

How did the 2014 passage of the Veterans Choice Program (VCP) affect prostate cancer care delivery in veterans?

**Findings:**

In this cohort study of 45 029 veterans with newly diagnosed prostate cancer, after VCP implementation, most patients elected to receive definitive treatment outside of the Veterans Health Administration system using purchased community care (CC). Patients treated in the purchased CC setting had a higher likelihood of receiving definitive treatment for low-risk prostate cancer.

**Meaning:**

Findings of this study suggest that there was an increase in access to prostate cancer care associated with the VCP; however, this access may come at the cost of lower-quality care (overtreatment) for low-risk prostate cancer.

## Teaser

This cohort study evaluates use of definitive treatments and conservative management at a community care vs Veterans Health Administration facility among veterans with prostate cancer.

## Introduction

The Veterans Choice Program (VCP) was enacted in 2014 to formalize access to specialized care by offering to purchase community care (CC) for veterans who were living more than 40 miles away from the nearest Veterans Health Administration (VHA) tertiary care facility (or 60-minute travel time), were unable to obtain an appointment within 30 days, or had a VHA tertiary care facility that was lacking necessary condition-specific resources.^1^ While non–VHA-purchased CC has always been available to veterans on a case-by-case basis, the VCP appears to have boosted its popularity, with 1.3 million veterans being authorized for non-VHA care in 2014, which increased to nearly 2.3 million in 2021.^2^ Purchased CC accounts for nearly 20% ($17.6 billion) of the total VHA clinical budget.^2^ Prostate cancer is the most commonly diagnosed solid organ tumor in male veterans, and an estimated one-quarter of veterans with newly diagnosed prostate cancer live in rural areas that meet the VCP distance eligibility criteria.^3^ Thus, a large percentage of veterans diagnosed with prostate cancer became eligible for VCP-funded purchased CC.

The VCP was enacted at a time when professional organizations began issuing guidelines that advised against aggressively treating clinically insignificant prostate cancer, as defined by early clinical stage, low Gleason score, and low prostate-specific antigen (PSA) level.^4,5^ While these changes represent a step toward decreasing the risks of overdiagnosis and overtreatment, implementation of these changes in clinical practice has varied by region and health care system.^6,7^

Within the VHA, conservative management of clinically insignificant prostate cancer has increased. From 2005 to 2015, there was a 2.3-fold increase (30% to 70%) in active surveillance and watchful waiting rates for prostate cancer deemed insignificant by National Comprehensive Cancer Network criteria; the increase was substantially higher than that observed in similar Medicare and community-based practice cohorts.^8,9,10^ The historically strong relationships between VHA medical centers and academic medical centers as well as the lack of volume-based financial incentive for VHA clinicians may be factors in the relatively higher rate of adherence to evidence-based guidelines, particularly those against performing aggressive procedures.^11^ However, it is unknown whether treatment patterns for veterans receiving prostate cancer urological care purchased with VCP funding differ from those for veterans receiving VHA care. Furthermore, although the VHA requires clinicians in the VCP to meet credentialing standards, the quality of VCP care is unknown and, to our knowledge, is not actively monitored by the VHA.^1,12,13^

The primary objective of this study was to describe the prevalence and patterns in VCP-funded purchased CC since the implementation of the VCP in 2014. We tested 2 hypotheses: (1) purchased CC would increase, particularly for veterans in rural areas who are disproportionately eligible, and (2) VHA care and purchased CC for prostate cancer would have similar quality.

## Methods

This retrospective cohort study focused on veterans diagnosed with prostate cancer between January 1, 2015 (the first full year of the VCP), and December 31, 2018 (the last full year of the VCP), before the changes brought on by the MISSION (Maintaining Internal Systems and Strengthening Integrated Outside Networks) Act, which expanded eligibility for purchased CC,^14^ or before standard practice patterns were altered by the COVID-19 pandemic.^15^ The VHA Iowa City Health System Institutional Review Board deemed the study exempt from review and waived the informed consent requirement and Health Insurance Portability and Accountability Act authorization because the study was a quality improvement project. We followed the Strengthening the Reporting of Observational Studies in Epidemiology (STROBE) reporting guideline.

### Data Sources

We obtained administrative data from 4 primary sources: (1) US Department of Veterans Affairs (VA) Corporate Data Warehouse, which contains data on outpatient visits, acute care stays, inpatient and outpatient diagnosis and procedure codes, prostate cancer–specific medication (eg, androgen deprivation therapy [ADT]) administration, and laboratory test results (eg, PSA level); (2) VA Oncology Module, which contains data on cancer diagnoses, histology, and pathology; (3) Planning Systems Support Group Geocoded Enrollment File, which contains information on patient residence, including rurality and distance to nearest VA facilities; and (4) VA Purchased Care Files, which contain details on care purchased by VHA from community-based facilities and practitioners, including the VA Program Integrity Tool files and VA Fee Basis files consisting of claims for services paid by VHA before implementation of the Program Integrity Tool system in 2018. Available care-related data included dates of service, inpatient and outpatient diagnoses, inpatient and outpatient procedure codes (eg, radical prostatectomy [RP], radiotherapy [RT], and prostate biopsy), and clinician details (eg, location and specialty).

### Study Cohort

We identified all patients with an initial diagnosis of prostate cancer between 2015 and 2018 in the VHA or purchased CC claims data using *International Classification of Diseases, Ninth Revision, Clinical Modification* diagnosis code 185.XX or *International Statistical Classification of Diseases, Tenth Revision, Clinical Modification* diagnosis code C61.xx. Patients were deemed to have a new diagnosis if they had 2 encounters for prostate cancer within 365 days and if they underwent a diagnostic prostate biopsy within approximately 180 days of the first appearance of the prostate cancer diagnosis. We excluded patients with fewer than 2 primary care VHA visits in the previous 2 years and 1 VHA visit in the 12 months after the prostate cancer diagnosis. Only regular users of the VHA health care system were included. The final cohort was followed up for prostate cancer–specific care through January 1, 2022 (eFigure in Supplement 1).

### Study Variables

Demographic variables included patient age, race and ethnicity, marital status, rural residence (urban, rural, or highly rural, the Rural-Urban Commuting Areas system used by the VHA Office of Rural Health), driving distance from residence to nearest VHA tertiary care facility, geographic region, annual income, Social Deprivation Index (SDI),^16^ and number of comorbidities (which was identified using VHA claims data for the 12 months prior to the initial diagnosis and was classified using the method of Quan et al^17^). Self-reported race and ethnicity data (Hispanic, non-Hispanic Black, non-Hispanic White, other racial and ethnic groups [including American Indian or Alaska Native, Asian, and Hawaii Native or Other Pacific Islander], and unknown) were obtained from VA enrollment files and analyzed in this study because of possible confounding between race and eligibility for purchased care.

Prostate cancer variables included Gleason grade group for a prostate biopsy (total Gleason score of ≤6 indicates grade group 1; total Gleason score of 7 indicates grade group 2 or 3; total Gleason score of ≥8 indicates grade group 4 or 5) and the most recent PSA level prior to prostate cancer diagnosis. Gleason scores of 3 + 4 and 4 + 3 could not be reliably differentiated due to limitations of the VHA pathology reporting methods. The VHA tertiary care facility variables included affiliation with academic centers and presence of RT facilities and/or availability of robotic assistance for prostate surgery at the assigned VHA tertiary care facility.

Initial definitive treatment was determined by documentation of any prostate cancer–related intervention within 18 months of diagnosis, categorized as surgery (open, laparoscopic, and robotic RP), RT (external beam and brachytherapy), or other definitive treatment (eg, cryotherapy and radiofrequency ablation). Conservative treatments were categorized as active surveillance (no evidence of definitive treatment after diagnosis with evidence of confirmatory and/or surveillance prostate biopsy within 18 months of diagnostic biopsy), watchful waiting (no evidence of definitive treatment, ADT or chemotherapy, or confirmatory or surveillance biopsy), or primary hormone therapy or ADT (antiandrogen and gonadotropin-releasing hormone [GnRH] agonist or antagonist only without evidence of definitive treatment). Any patients who received multiple therapies (eg, adjuvant RT after surgery, salvage RP after RT, salvage cryotherapy, and ADT with RT) were categorized by their initial primary treatment.

The location of prostate cancer care was determined from coding and billing data, and any non-VHA prostate cancer urological care paid for with VCP funding was considered to be purchased CC. Definitive treatment that was received outside of the VHA or not purchased through the VCP was not identified for this study.

### Statistical Analysis

We conducted descriptive analyses of the overall cohort and then the location (VHA vs purchased CC) at which most postdiagnostic prostate cancer urological care was performed. We ascertained the percentage of definitive treatment (eg, RP and RT) and active surveillance performed outside of the VHA facility stratified by urological care location period. We used these analyses to describe the association between distance from VHA tertiary care facility and prostate cancer care location in the years after VCP implementation.

We identified factors associated with receiving definitive treatment for prostate cancer, adjusting for all demographic characteristics, prostate cancer characteristics, and VHA tertiary care facility variables. Although we could not specifically determine the prostate cancer care location or clinician that was most responsible for the ultimate treatment decision, we ultimately assigned patients to either the purchased CC or VHA location based on 3 separate factors: (1) location of their diagnostic biopsy, (2) location of most of their postdiagnostic PSA laboratory testing, and (3) location of most of their postdiagnostic urological care encounters. For example, with factor 3, a patient who obtained RT outside of the VHA (a common occurrence given that most VHA centers do not provide RT) but saw urologists within the VHA was labeled as a primary VHA user despite being treated at a non-VHA facility. Using multivariable models with a log link and Poisson distribution to estimate relative risk (RR) ratios, we calculated the relative likelihood of definitive treatment for patients treated by purchased CC vs VHA. Models controlled for diagnosis year, age, race and ethnicity, number of comorbidities, area SDI, Gleason grade group, PSA category (<4.0, 4.0 to <10.0, or ≥10.0 ng/mL, missing data; to convert PSA level to microgram per liter, multiply by 1.0), and treatment options available at the patient’s primary VA facility (robotic-assisted surgery, RT, or neither). Models were estimated as generalized estimating equations to control for the clustering of veterans within the primary VA facility.

Additionally, to determine the association between purchased CC and treatment quality, we examined how location affected definitive treatment for low-risk prostate cancer, defined as a diagnostic biopsy showing Gleason grade group 1 prostate cancer (Gleason scores of 3 + 3 or lower). This quality measure was based on the 2022 American Urological Association/American Society for Radiation Oncology Clinically Localized Prostate Cancer guideline 10, which states that “For patients with low-risk prostate cancer, clinicians should recommend [active surveillance] as the preferred management option.”^4^^(p14)^ The rationale behind this guideline is that Gleason grade group 1 prostate cancer has a low likelihood of progression and death from disease,^18,19^ which is supported by low rates of metastatic disease (<2%) and prostate cancer–specific mortality (<1%) with Gleason grade group 1 disease on active surveillance.^20,21^

Two-sided *P* < .05 indicated statistical significance. All analyses were performed with SAS, version 9 (SAS Institute Inc), from March to July 2023.

## Results

The cohort included 45 029 male veterans (mean [SD] age, 67.1 [6.9] years) with newly diagnosed prostate cancer. Of these patients, 64.1% underwent definitive treatment, with 21.6% undergoing RP, 42.1% undergoing RT, and 0.4% undergoing cryotherapy (Table 1). Among those without evidence of definitive treatment, 8.3% were managed with active surveillance and 21.4% were presumed to have received watchful waiting (or care not paid for with VHA or VCP funding). Over the study period, 37.5% of patients received RP and 66.7% received RT at non-VHA facilities with purchased CC, whereas most active surveillance management (92.5% of patients) remained within the VHA.

**Table 1.** Patient and Facility Characteristics Overall and Stratified by Location of Most Postdiagnostic Prostate Cancer Urological Care

Variable	Patients, No. (%)			*P* value
	Overall	VHA	Purchased CC	
Total	45 029 (100)	35 483 (78.8)	9546 (21.2)	
Age, mean (SD), y	67.1 (6.9)	67.1 (6.8)	66.8 (7.0)	NA
Age, y				
<55	2303 (5.1)	1731 (4.9)	572 (6.0)	<.001
55-59	4523 (10.0)	3555 (10.0)	968 (10.1)	
60-64	8156 (18.1)	6423 (18.1)	1733 (18.2)	
65-69	16 184 (35.9)	12 860 (36.2)	3324 (34.8)	
70-74	9784 (21.7)	7659 (21.6)	2125 (22.3)	
≥75	4079 (9.1)	3255 (9.2)	824 (8.6)	
Rural residence				
Urban	28 994 (64.3)	23 882 (67.3)	5112 (53.6)	<.001
Rural	15 361 (34.1)	11 137 (31.4)	4224 (44.3)	
Highly rural	674 (1.5)	464 (1.3)	210 (2.2)	
Race and ethnicity^a^				
Non-Hispanic Black	14 338 (31.8)	11 925 (33.6)	2413 (25.3)	<.001
Non-Hispanic White	26 943 (59.8)	20 661 (58.2)	6282 (65.8)	
Other^b^	2473 (5.5)	1918 (5.4)	955 (5.8)	
Unknown	1175 (2.6)	979 (2.8)	296 (3.1)	
Marital status				
Divorced	14211 (31.6)	11 310 (31.9)	2901 (30.4)	<.001
Married	23 928 (53.1)	18 584 (52.4)	5344 (56.0)	
Single	4540 (10.1)	3700 (10.4)	840 (8.8)	
Widowed	2179 (4.8)	1765 (5.0)	414 (4.3)	
Unknown	171 (0.3)	124 (0.4)	47 (0.5)	
Geographic region				
Northeast	5119 (11.4)	4420 (12.5)	699 (7.3)	<.001
South	21 592 (48.0)	16 838 (47.5)	4754 (49.8)	
Midwest	10 361 (23.0)	8009 (22.6)	2352 (24.6)	
West	7957 (17.7)	6215 (17.5)	1735 (18.2)	
Driving distance to VHA tertiary care facility, mean (SD), miles	91.1 (85.6)	79.5 (81.3)	134.1 (112.0)	<.001
No. of comorbidities				
0	5878 (13.1)	3161 (8.9)	2717 (28.5)	<.001
1	4126 (9.2)	3374 (9.5)	752 (7.9)	
≥2	35 025 (77.8)	28 948 (81.6)	6099 (63.7)	
SDI (SD)	55.2 (2.6)	5.56 (2.7)	5.40 (2.2)	NA
Year of diagnosis				
2015	8921 (19.8)	7402 (20.9)	1519 (15.9)	<.001
2016	10 009 (22.2)	8384 (23.6)	1625 (17.0)	
2017	12 077 (26.8)	9414 (26.5)	2663 (27.9)	
2018	14 022 (31.1)	10 283 (29.0)	3739 (39.2)	
VHA tertiary care facility characteristics				
Academic center affiliation	41 245 (91.6)	32 452 (91.5)	8793 (92.1)	.04
RT only	9827 (21.8)	7771 (21.9)	2056 (21.5)	<.001
Robotic-assisted surgery, with or without RT	18 351 (40.8)	16 360 (46.1)	1991 (20.9)	

Abbreviations: CC, community care; NA, not applicable; RT, radiotherapy; SDI, Social Deprivation Index; VA, US Department of Veterans Affairs; VHA, Veterans Health Administration.

^a^ Race and ethnicity were self-reported and obtained from VA enrollment files.

^b^ Other categories included Hispanic and other racial and ethnic groups, including American Indian or Alaska Native, Asian, and Hawaii Native or Other Pacific Islander.

Tables 1 and 2 show patient, facility, and demographic characteristics of the study cohort stratified by the location at which most of their postdiagnostic urological care was received. Notable demographic differences were as follows: patients receiving purchased CC compared with those receiving VHA care were more likely to live in rural settings (44.3% vs 31.4%), live farther from their VHA tertiary care facility (134.1 vs 79.5 miles), have fewer comorbidities (≥2: 63.7% vs 81.6%), and be assigned to a VHA tertiary care facility without robotic assistance capabilities (20.9% vs 46.1%) (Table 1). Notable tumor and treatment differences included the following: patients receiving purchased CC compared with those receiving VHA care were more likely to get their biopsy outside of the VHA (53.2% vs 3.1%), less likely to have their pathology results in the VA Oncology Module (45.9% vs 81.6%), more likely to undergo definitive treatment (77.2% vs 60.6%), and more likely to get definitive treatment at a non-VHA location (93.2% vs 44.4%) (Table 2). Overall, 56.4% of all veterans diagnosed with prostate cancer during the study period received purchased CC.

**Table 2.** Prostate Cancer and Treatment Characteristics Overall and at Veterans Health Administration (VHA), the Location of Most Postdiagnostic Urological Care^a^

Variable	Patients, No. (%)			*P* value
	Overall	VHA	Purchased CC	
Baseline PSA category, ng/mL				
<4.0	3407 (7.6)	2653 (7.5)	754 (7.9)	.006
4.0 to <10.0	20 907 (46.4)	16 368 (46.1)	4539 (47.6)	
≥10.0	9395 (20.9)	7427 (20.9)	1968 (20.6)	
Missing data	11 320 (25.1)	9035 (25.5)	2285 (23.9)	
Diagnostic biopsy location				
VHA facility	38 866 (86.3)	34 398 (97.0)	4468 (46.8)	<.001
Non-VHA facility	6163 (13.7)	1085 (3.1)	5078 (53.2)	
VA Oncology Module data available	33 321 (74.0)	28 942 (81.6)	4379 (45.9)	<.001
Gleason score				
≤6: Grade group 1	10 417 (31.3)	9180 (31.7)	1237 (28.3)	<.001
7: Grade group 2 or 3	14 727 (44.2)	12 659 (43.7)	2068 (47.2)	
≥8: Grade group 4 or 5	8177 (24.5)	7103 (24.6)	1074 (24.5)	
Definitive treatment				
Overall	28 866 (64.1)	21 496 (60.6)	7370 (77.2)	<.001
Surgery	9703 (21.6)	6660 (18.8)	3043 (31.9)	<.001
RT	18 963 (42.1)	14 747 (41.6)	4216 (44.2)	<.001
Cryotherapy	200 (0.4)	89 (0.3)	111 (1.2)	<.001
Conservative management				
Active surveillance	3733 (8.3)	3348 (9.4)	385 (4.0)	<.001
Watchful waiting	9619 (21.4)	8273 (23.3)	1346 (14.1)	<.001
Primary hormone therapy or ADT	2811 (6.2)	2366 (6.7)	445 (4.7)	<.001
Definitive treatment location				
VHA facility	12 457 (43.2)	11 958 (55.6)	499 (6.8)	<.001
Non-VHA facility	16 409 (56.8)	9538 (44.4)	6871 (93.2)	

Abbreviations: ADT, androgen deprivation therapy; CC, community care; PSA, prostate-specific antigen; RT, radiotherapy; VA, US Department of Veterans Affairs.

SI conversion factor: To convert PSA level from nanogram per milliliter to microgram per liter, multiply by 1.0.

^a^ Prostate cancer was diagnosed between 2015 and 2018.

The absolute number of patients with a newly diagnosed prostate cancer increased over the study period from 8921 in 2015 to 14 022 in 2018 (Figure, A), although the overall percentage of patients electing definitive care remained relatively stable from 62.4% in 2015 to 63.5% in 2018. The percentage of patients electing definitive treatment with purchased CC care increased each study year (Figure, B) from 46.8% in 2015 to 62.7% in 2018, with the absolute number of patients undergoing purchased CC surgery increasing nearly 3 times (from 521 to 1346) and purchased CC RT increasing over 2 times (from 2076 to 4204).

**Figure.**
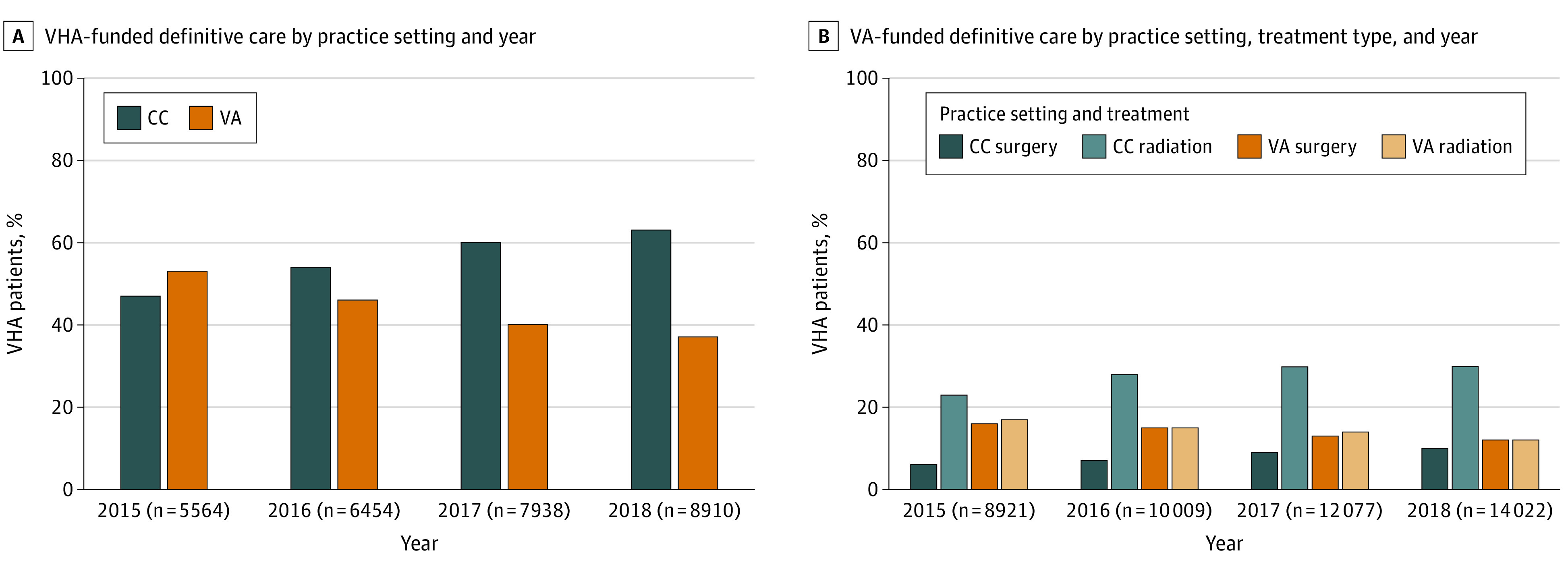
Definitive Care by Practice Setting, Treatment Type, and Year CC indicates community care; VA, US Department of Veterans Affairs; VHA, Veterans Health Administration.

Table 3 shows a significantly higher likelihood of receiving definitive treatment when prostate cancer treatment decisions were made in the purchased CC setting (adjusted RR ratio, 1.27; 95% CI, 1.24-1.31). Full models are provided in eTables 1 to 6 in Supplement 1.

**Table 3.** Unadjusted Treatment Rates and Adjusted Models Assessing Association of Receiving Definitive Treatment With Location

Model variable	Unadjusted treatment rates, No. (%)		Adjusted RR of treatment for purchased CC vs VHA^a^	
	Purchased CC	VHA	RR ratio (95% CI)	*P* value
Initial biopsy performed in CC^b^	4237 (73.5)	24 629 (67.6)	1.16 (1.12-1.20)	<.001
Most postdiagnostic PSA testing performed in CC^c^	4961 (87.4)	23 905 (65.4)	1.32 (1.29-1.35)	<.001
Most postdiagnostic urological care encounters in CC^d^	7370 (81.0)	21 496 (64.9)	1.27 (1.24-1.31)	<.001

Abbreviations: CC, community care; PSA, prostate-specific antigen; RR, relative risk; VHA, Veterans Health Administration.

^a^ Controlling for year of diagnosis, age, race and ethnicity, distance from VHA tertiary care facility, Gleason grade group, number of comorbidities (classified using the Quan et al^17^ method), Social Deprivation Index, PSA level, and facility resources.

^b^ See eTable 1 in Supplement 1.

^c^ See eTable 2 in Supplement 1.

^d^ See eTable 3 in Supplement 1.

Table 4 shows that, in a subcohort of 10 248 patients with Gleason grade group 1 prostate cancer, 39.8% underwent definitive treatment. Unadjusted and adjusted definitive treatment rates were significantly higher when care decisions were made in the purchased CC vs VHA setting (adjusted RR ratio, 1.79; 95% CI, 1.65-1.93).

**Table 4.** Unadjusted and Adjusted Models Assessing Association of Receiving Definitive Treatment With Location Among 10 248 Patients With Gleason Grade Group 1 Prostate Cancer Eligible for Conservative Management

Model variable	Unadjusted treatment rates, No. (%)		Adjusted RR of treatment for purchased CC vs VHA^a^	
	Purchased CC	VHA	RR ratio (95% CI)	*P* value
Initial biopsy performed in CC^b^	283 (50.5)	3793 (39.2)	1.32 (1.18-1.46)	<.001
Most postdiagnostic PSA testing performed in CC^c^	457 (76.7)	3619 (37.5)	1.93 (1.81-2.07)	<.001
Most postdiagnostic urological care encounters in CC^d^	802 (65.9)	3274 (36.3)	1.79 (1.65-1.93)	<.001

Abbreviations: CC, community care; PSA, prostate-specific antigen; RR, relative risk; VHA, Veterans Health Administration.

^a^ Controlling for year of diagnosis, age, race and ethnicity, distance from VHA tertiary care facility, Gleason Grade Group, number of comorbidities (classified using the Quan et al^17^ method), Social Deprivation Index, PSA level, and facility resources.

^b^ See eTable 4 in Supplement 1.

^c^ See eTable 5 in Supplement 1.

^d^ See eTable 6 in Supplement 1.

## Discussion

In this population-based cohort study, we found that over half of all definitive treatment for prostate cancer is now being provided in non-VHA facilities using VCP funding. The shift to CC after VCP implementation was most pronounced for surgical care. Contrary to our second hypothesis, we found differences between VHA and purchased CC in prostate cancer treatment quality. The relative risk of receiving definitive treatment for low-risk prostate cancer was 1.79 for patients receiving their urological care outside of the VHA with VCP-funded purchased CC vs VHA care.

Because prostate cancer is one of the more common solid organ cancers diagnosed in veterans, providing broad access to cancer specialists has historically challenged the VHA system. Accordingly, ensuring that veterans received timely specialized care consultations was one of the primary reasons for creating the VCP.^1,13^ Early reports on the VCP identified care coordination concerns by both clinicians and patients.^1,12,13^ However, overall wait times have decreased and patient satisfaction with the program is high, validating its importance and utility.^12,22^ Findings of the present study confirmed that the VCP has succeeded in its primary goal of improving access to specialized care, especially for veterans in rural areas. However, to our knowledge, this study is the first to argue that this improved access may come at the cost of lower quality. Other surgical outcome studies have shown similar rates of complications for other high-volume purchased CC procedures (eg, cataract surgery and total knee arthroplasty), suggesting similar surgical safety profiles.^23,24^ By focusing on the appropriateness of prostate cancer treatment as a quality measure and not on treatment outcomes, we highlighted that a potential weakness of the VCP may be the risk for overtreatment.

There are 3 explanations for these findings that have potential policy implications and thus deserve attention. First, the financial incentives of the VCP for clinicians outside the VHA must be considered. Although accepting patients in the program requires the clinician to accept Medicare reimbursement rates, which may make VCP-funded surgical care less costly than surgery within the VHA,^25^ providing any intervention will nearly always be more financially rewarding to the community practice than providing no care.^26^ With the multitude of options available to patients and clinicians, financial incentives have been shown to play a role in prostate cancer care in nonintegrated systems, often toward more aggressive therapy.^27^ Second, fragmentation is inherent to purchased CC. As has been shown previously, clinicians outside of the VHA system may view their relationship with the VHA patient as contractual, and both the patient and clinician may be motivated to undergo definitive treatment in the limited time allotted in the contract.^28^ Similarly, active surveillance, while recommended for most low-risk prostate cancer, can be a multiyear process with yearly biopsies and PSA testing.^29^ Additionally, discussions about not immediately treating a cancer can be complex, time-consuming, and confusing, and selecting active surveillance requires the patient to trust a physician’s recommendation that it is a beneficial and safe strategy.^9^ Although VCP contracts can be renewed yearly, the need for long-term care and reassuring patients about the appropriateness of active surveillance may pose a logistical challenge that incentivizes intervention. Third, the penetrance of evidence-based medicine and best-practice policies is historically higher in VHA facilities, which are often affiliated with academic centers.^8^

### Limitations

This study has several limitations, including alternative explanations for the discrepancy in care quality. First, patient characteristics that were not accounted for in the models may be responsible for some of the differences seen. While we did control for age, number of comorbidities, and SDI, patients who seek care outside of the VHA are known to be healthier than those who seek care inside of the VHA,^30^ and healthier patients are more likely to gain benefit from definitive treatment than those with lower life expectancies.^31^ Although the American Urological Association/American Society for Radiation Oncology guidelines recommend active surveillance for Gleason grade group 1 prostate cancer without consideration of patient age, the decision is ultimately the patient’s, and many males, especially those with longer life expectancies, are disproportionately challenged by the concept of not treating their cancer.^32^ Second, the pathology data for this cohort were incomplete. The models did adjust for missing pathology data, and there were similar distributions of Gleason grade groups among patients with known pathologies between the purchased CC and VHA cohorts. Nevertheless, unmeasured differences in histopathology may partially account for treatment differences. For example, VA Oncology Module data do not provide the percentage of cores with Gleason grade group 1 pathology, a factor known to affect treatment decisions in both patients and clinicians.

Third, we included only regular users of the VHA, a population known to have a higher number of comorbidities than the general veteran population.^30^ Fourth, we did not review Medicare claims for definitive treatment outside of the VHA and the VCP. Fifth, this study was a retrospective review of administrative data; therefore, the exact decision-making processes regarding where and how to treat individual prostate cancer cases were unknown, requiring us to create indirect care decision location variables to test the study hypotheses.

## Conclusions

In the years following the 2014 implementation of the VCP, the percentage of veterans electing VCP-funded purchased CC over VHA care continued to increase to the point where most of all definitive treatments today are performed outside of VHA facilities. While this finding is favorable regarding meeting the VCP goal of improving access to specialized care, it suggests that increased access may come at the cost of low care quality (overtreatment) for low-risk prostate cancer.

## Notes

Supplement 1. **eTable 1.** Multivariable Model Assessing Risk of Definitive Prostate Cancer Treatment (Surgery, Radiation or Cryotherapy) for Prostate Cancer (All Grades). Variable of Interest: Location of the Diagnostic Biopsy **eTable 2.** Multivariable Model Assessing Risk of Definitive Prostate Cancer Treatment (Surgery, Radiation or Cryotherapy) for Prostate Cancer (All Grades). Variable of Interest: Location of the Majority of Post-Prostate Cancer Diagnosis PSA Testing **eTable 3.** Multivariable Model Assessing Risk of Definitive Prostate Cancer Treatment (Surgery, Radiation or Cryotherapy) for Prostate Cancer (All Grades). Variable of Interest: Location of the Majority of Post-Prostate Cancer Diagnosis Urologic Care **eTable 4.** Multivariable Model Assessing Risk of Definitive Prostate Cancer Treatment (Surgery, Radiation or Cryotherapy) for Grade Group 1 Prostate Cancer. Variable of Interest: Location of the Diagnostic Biopsy **eTable 5.** Multivariable Model Assessing Risk of Definitive Prostate Cancer Treatment (Surgery, Radiation or Cryotherapy) for Grade Group 1 Prostate Cancer. Variable of Interest: Location of the Majority of Post-Prostate Cancer Diagnosis PSA Testing **eTable 6.** Multivariable Model Assessing Risk of Definitive Prostate Cancer Treatment (Surgery, Radiation or Cryotherapy) for Grade Group 1 Prostate Cancer. Variable of Interest: Location of the Majority of Post-Prostate Cancer Diagnosis Urologic Care **eFigure.** Cohort Creation of Newly Diagnosed Prostate Cancer Patients That Are Regular Users of the VHA With Known Location of Residence

Supplement 2.
Data Sharing Statement
