# Supplementary material for: Location and Types of Treatment for Prostate Cancer After the Veterans Choice Program Implementation
Source: JAMA Netw Open. 2023 Oct 19;6(10):e2338326. doi: 10.1001/jamanetworkopen.2023.38326 (PMC10587787; doi:10.1001/jamanetworkopen.2023.38326)
Supplement: Supplement 1. — eTable 1. Multivariable Model Assessing Risk of Definitive Prostate Cancer Treatment (Surgery, Radiation or Cryotherapy) for Prostate Cancer (All Grades). Variable of Interest: Location of the Diagnostic Biopsy eTable 2. Multivariable Model Assessing Risk of Definitive Prostate Cancer Treatment (Surgery, Radiation or Cryotherapy) for Prostate Cancer (All Grades). Variable of Interest: Location of the Majority of Post-Prostate Cancer Diagnosis PSA Testing eTable 3. Multivariable Model Assessing Risk of Definitive Prostate Cancer Treatment (Surgery, Radiation or Cryotherapy) for Prostate Cancer (All Grades). Variable of Interest: Location of the Majority of Post-Prostate Cancer Diagnosis Urologic Care eTable 4. Multivariable Model Assessing Risk of Definitive Prostate Cancer Treatment (Surgery, Radiation or Cryotherapy) for Grade Group 1 Prostate Cancer. Variable of Interest: Location of the Diagnostic Biopsy eTable 5. Multivariable Model Assessing Risk of Definitive Prostate Cancer Treatment (Surgery, Radiation or Cryotherapy) for Grade Group 1 Prostate Cancer. Variable of Interest: Location of the Majority of Post-Prostate Cancer Diagnosis PSA Testing eTable 6. Multivariable Model Assessing Risk of Definitive Prostate Cancer Treatment (Surgery, Radiation or Cryotherapy) for Grade Group 1 Prostate Cancer. Variable of Interest: Location of the Majority of Post-Prostate Cancer Diagnosis Urologic Care eFigure. Cohort Creation of Newly Diagnosed Prostate Cancer Patients That Are Regular Users of the VHA With Known Location of Residence [file jamanetwopen-e2338326-s001.pdf]

Supplemental Online Content

Erickson BA, Hoffman RM, Wachsmuth J, Packiam VT, Vaughan-Sarrazin M. Location and types of treatment for prostate cancer after the Veterans Choice Program implementation. *JAMA Netw Open*. 2023;6(10):e2338326. doi:10.1001/jamanetworkopen.2023.38326

eTable 1. Multivariable Model Assessing Risk of Definitive Prostate Cancer Treatment (Surgery, Radiation or Cryotherapy) for Prostate Cancer (All Grades). Variable of Interest: Location of the Diagnostic Biopsy
eTable 2. Multivariable Model Assessing Risk of Definitive Prostate Cancer Treatment (Surgery, Radiation or Cryotherapy) for Prostate Cancer (All Grades). Variable of Interest: Location of the Majority of Post-Prostate Cancer Diagnosis PSA Testing
eTable 3. Multivariable Model Assessing Risk of Definitive Prostate Cancer Treatment (Surgery, Radiation or Cryotherapy) for Prostate Cancer (All Grades). Variable of Interest: Location of the Majority of Post-Prostate Cancer Diagnosis Urologic Care
eTable 4. Multivariable Model Assessing Risk of Definitive Prostate Cancer Treatment (Surgery, Radiation or Cryotherapy) for Grade Group 1 Prostate Cancer. Variable of Interest: Location of the Diagnostic Biopsy
eTable 5. Multivariable Model Assessing Risk of Definitive Prostate Cancer Treatment (Surgery, Radiation or Cryotherapy) for Grade Group 1 Prostate Cancer. Variable of Interest: Location of the Majority of Post-Prostate Cancer Diagnosis PSA Testing
eTable 6. Multivariable Model Assessing Risk of Definitive Prostate Cancer Treatment (Surgery, Radiation or Cryotherapy) for Grade Group 1 Prostate Cancer. Variable of Interest: Location of the Majority of Post-Prostate Cancer Diagnosis Urologic Care
eFigure. Cohort Creation of Newly Diagnosed Prostate Cancer Patients That Are Regular Users of the VHA With Known Location of Residence

This supplemental material has been provided by the authors to give readers additional information about their work.

eTable 1. Multivariable Model Assessing Risk of Definitive Prostate Cancer Treatment (Surgery, Radiation or Cryotherapy) for Prostate Cancer (all grades). Variable of Interest: Location of the Diagnostic Biopsy.

Variable		RR	Lower CI	Upper CI	p-value
Intercept		0.58	0.53	0.64	<0.0001
Biopsy Site	CC	1.16	1.12	1.20	<0.0001
	VA	Ref			
Dx Year	2016	1.03	1.01	1.06	0.0035
	2017	1.05	1.02	1.07	<0.0001
	2018	1.02	1.00	1.05	0.11
	2015	Ref			
Age		0.99	0.99	0.99	<0.0001
Race	Black (non-Hisp)	1.00	0.99	1.01	0.76
	Other	0.99	0.96	1.01	0.33
	Unknown	0.96	0.93	0.99	0.03
	White (non-Hisp)	Ref			
Distance to Nearest VTCF	>90 miles	1.03	1.00	1.05	0.02
	40 – 90 miles	1.02	1.00	1.04	0.09
	0 – 40 miles	Ref			
Gleason Group	Grade Group 2/3	2.02	1.92	2.12	<0.0001
	Grade Group 4/5	2.14	2.04	2.25	<0.0001
	Unknown	1.53	1.46	1.61	<0.0001
Sum Comorbidity		1.00	1.00	1.00	0.03
SDI Score		1.01	1.01	1.01	<0.0001
PSA Category	4 to 10	1.04	1.01	1.07	0.013
	>10	1.15	1.11	1.19	<0.0001
	Missing	1.05	1.01	1.09	0.02
	<4	Ref			
Facility	Robot Capability	1.03	1.00	1.05	0.06
	Radiation Only	1.03	0.99	1.06	0.10
	Neither	Ref			

© 2023 Erickson BA et al. *JAMA Network Open*

eTable 2. Multivariable Model Assessing Risk of Definitive Prostate Cancer Treatment (Surgery, Radiation or Cryotherapy) for Prostate Cancer (all grades). Variable of Interest: Location of the Majority of Post-Prostate Cancer Diagnosis PSA Testing

Variable		RR	Lower CI	Upper CI	p-value
Intercept		0.58	0.53	0.64	<0.0001
Post-Diagnosis PSA	CC	1.32	1.29	1.35	<0.0001
	VA	Ref			
Dx Year	2016	1.03	1.01	1.05	0.02
	2017	1.03	1.01	1.06	0.0031
	2018	1.01	0.98	1.04	0.59
	2015	Ref			
Age		0.99	0.99	0.99	<0.0001
Race	Black (non-Hisp)	1.00	0.99	1.02	0.72
	Other	0.99	0.96	1.01	0.33
	Unknown	0.96	0.93	1.00	0.03
	White (non-Hisp)	Ref			
Distance to Nearest VTCF	>90 miles	1.01	0.98	1.03	0.64
	40 – 90 miles	1.01	0.98	1.03	0.49
	0 – 40 miles	Ref			
Gleason Group	Grade Group 2/3	2.00	1.90	2.09	<0.0001
	Grade Group 4/5	2.11	2.01	2.22	<0.0001
	Unknown	1.53	1.46	1.61	<0.0001
Sum Comorbidity		1.00	1.00	1.00	0.14
SDI Score		1.01	1.01	1.01	<0.0001
PSA Category	4 to 10	1.04	1.01	1.08	0.007
	>10	1.15	1.11	1.20	<0.0001
	Missing	1.05	1.01	1.09	0.02
	<4	Ref			
Facility	Robot Capability	1.03	1.00	1.06	0.02
	Radiation Only	1.03	0.99	1.06	0.11
	Neither	Ref			

© 2023 Erickson BA et al. *JAMA Network Open*

eTable 3. Multivariable Model Assessing Risk of Definitive Prostate Cancer Treatment (Surgery, Radiation or Cryotherapy) for Prostate Cancer (all grades). Variable of Interest: Location of the Majority of Post-Prostate Cancer Diagnosis Urologic Care

Variable		RR	Lower CI	Upper CI	p-value
Intercept		0.65	0.58	0.72	<0.0001
Post-Diagnosis Urology (majority)	CC	1.27	1.23	1.31	<0.0001
	VA	Ref			
Dx Year	2016	1.00	0.98	1.02	0.85
	2017	1.00	0.97	1.02	0.78
	2018	0.97	0.94	1.00	0.05
	2015	Ref			
Age		0.99	0.99	0.99	<0.0001
Race	Black (non-Hisp)	1.01	0.99	1.03	0.21
	Other	0.99	0.96	1.03	0.77
	Unknown	0.97	0.92	1.01	0.14
	White (non-Hisp)	Ref			
Distance to Nearest VTCF	>90 miles	0.98	0.96	1.01	0.26
	40 – 90 miles	0.99	0.97	1.02	0.55
	0 – 40 miles	Ref			
Gleason Group	Grade Group 2/3	1.82	1.73	1.92	<0.0001
	Grade Group 4/5	1.92	1.83	2.01	<0.0001
	Unknown	1.39	1.22	1.47	<0.0001
Sum Comorbidity		1.00	1.00	1.00	0.008
SDI Score		1.01	1.00	1.01	<0.0001
PSA Category	4 to 10	1.10	1.06	1.13	<0.0001
	>10	1.19	1.15	1.24	<0.0001
	Missing	1.08	1.03	1.13	0.0004
	<4	Ref			
Facility	Robot Capability	1.03	1.00	1.05	0.08
	Radiation Only	1.01	0.98	1.04	0.64
	Neither	Ref			

© 2023 Erickson BA et al. *JAMA Network Open*

eTable 4. Multivariable Model Assessing Risk of Definitive Prostate Cancer Treatment (Surgery, Radiation or Cryotherapy) for Grade Group 1 Prostate Cancer. Variable of Interest: Location of the Diagnostic Biopsy

Variable		RR	Lower CI	Upper CI	p-value
Intercept		1.02	0.79	1.32	0.87
Biopsy Site	CC	1.32	1.19	1.46	<0.0001
	VA	Ref			
Dx Year	2016	1.04	0.97	1.12	0.29
	2017	1.09	1.01	1.18	0.02
	2018	1.00	0.92	1.09	1.00
	2015	Ref			
Age		0.98	0.98	0.98	<0.0001
Race	Black (non-Hisp)	1.09	1.04	1.15	0.001
	Other	1.02	0.94	1.12	0.58
	Unknown	1.00	0.87	1.15	0.96
	White (non-Hisp)	Ref			
Distance to Nearest VTCF	>90 miles	1.11	1.02	1.20	0.02
	40 – 90 miles	1.14	1.06	1.23	0.0004
	0 – 40 miles	Ref			
Sum Comorbidity		0.99	0.98	1.00	0.06
SDI Score		1.00	1.00	1.01	0.23
PSA Category	4 to 10	1.09	1.01	1.18	0.04
	>10	1.54	1.41	1.68	<0.0001
	Missing	1.15	1.03	1.28	0.01
	<4	Ref			
Facility	Robot Capability	1.03	0.95	1.12	0.51
	Radiation Only	1.03	0.94	1.12	0.57
	Neither	Ref			

© 2023 Erickson BA et al. *JAMA Network Open*

eTable 5. Multivariable Model Assessing Risk of Definitive Prostate Cancer Treatment (Surgery, Radiation or Cryotherapy) for Grade Group 1 Prostate Cancer. Variable of Interest: Location of the Majority of Post-Prostate Cancer Diagnosis PSA Testing

Variable		RR	Lower CI	Upper CI	p-value
Intercept		0.98	0.76	1.26	0.85
Post-Diagnosis PSA	CC	1.94	1.81	2.07	<0.0001
	VA	Ref			
Dx Year	2016	1.03	0.96	1.11	0.42
	2017	1.07	0.99	1.16	0.08
	2018	0.97	0.90	1.06	0.54
	2015	Ref			
Age		0.98	0.98	0.99	<0.0001
Race	Black (non-Hisp)	1.07	1.02	1.13	0.009
	Other	1.01	0.93	1.10	0.76
	Unknown	0.97	0.85	1.11	0.64
	White (non-Hisp)	Ref			
Distance to Nearest VTCF	>90 miles	1.06	0.97	1.14	0.20
	40 – 90 miles	1.11	1.03	1.20	0.005
	0 – 40 miles	Ref			
Sum Comorbidity		0.99	0.98	1.00	0.13
SDI Score		1.01	1.00	1.01	0.19
PSA Category	4 to 10	1.08	1.00	1.18	0.06
	>10	1.52	1.29	1.67	<0.0001
	Missing	1.13	1.02	1.26	0.02
	<4	Ref			
Facility	Robot Capability	1.05	0.96	1.14	0.29
	Radiation Only	1.02	0.94	1.12	0.63
	Neither	Ref			

© 2023 Erickson BA et al. *JAMA Network Open*

eTable 6. Multivariable Model Assessing Risk of Definitive Prostate Cancer Treatment (Surgery, Radiation or Cryotherapy) for Grade Group 1 Prostate Cancer. Variable of Interest: Location of the Majority of Post-Prostate Cancer Diagnosis Urologic Care

Variable		RR	Lower CI	Upper CI	p-value
Intercept		0.88	0.68	1.14	0.34
Post-Diagnosis Urology (majority)	CC	1.79	1.65	1.93	<0.0001
	VA	Ref			
Dx Year	2016	1.04	0.97	1.12	0.31
	2017	1.07	0.99	1.15	0.09
	2018	0.96	0.89	1.04	0.35
	2015	Ref			
Age		0.98	0.98	0.99	<0.0001
Race	Black (non-Hisp)	1.09	1.04	1.16	0.001
	Other	1.02	0.94	1.12	0.62
	Unknown	1.01	0.99	1.16	0.88
	White (non-Hisp)	Ref			
Distance to Nearest VTCF	>90 miles	1.06	0.97	1.15	0.22
	40 – 90 miles	1.14	1.05	1.22	0.0008
	0 – 40 miles	Ref			
Sum Comorbidity		1.00	0.99	1.00	0.31
SDI Score		1.01	1.00	1.01	0.17
PSA Category	4 to 10	1.11	1.02	1.20	0.02
	>10	1.52	1.29	1.67	<0.0001
	Missing	1.16	1.04	1.29	0.01
	<4	Ref			
Facility	Robot Capability	1.05	0.97	1.14	0.20
	Radiation Only	1.02	0.93	1.12	0.65
	Neither	Ref			

© 2023 Erickson BA et al. *JAMA Network Open*

Appendix 1: Cohort Creation of Newly Diagnosed Prostate Cancer Patients that are Regular Users of the VHA with Known Location of Residence

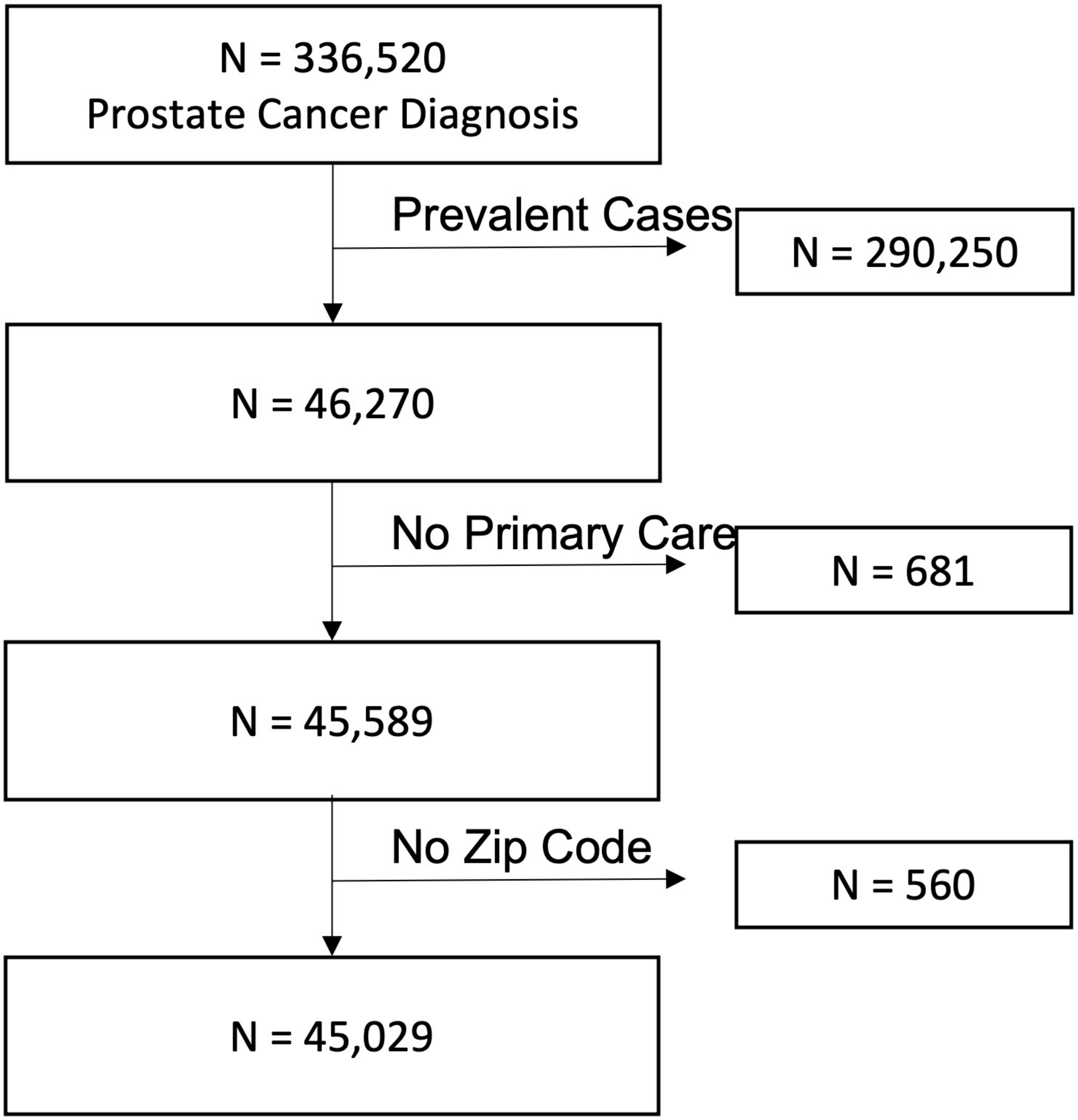

© 2023 Erickson BA et al. *JAMA Network Open*
