# Supplementary material for: Location and Types of Treatment for Prostate Cancer After the Veterans Choice Program Implementation
Source: JAMA Netw Open. 2023 Oct 19;6(10):e2338326. doi: 10.1001/jamanetworkopen.2023.38326 (PMC10587787; doi:10.1001/jamanetworkopen.2023.38326)
Supplement: Supplement 2. — Data Sharing Statement [file jamanetwopen-e2338326-s002.pdf]

Data Sharing Statement

Erickson. Location and Types of Treatment for Prostate Cancer After the Veterans Choice Program Implementation. *JAMA Netw Open*. Published October 19, 2023. doi:10.1001/jamanetworkopen.2023.38326

Data
Data available: No

Additional Information
Explanation for why data not available: VHA data
